# The Multi-Kinase Inhibitor RepSox Enforces Barrier Function in the Face of Both VEGF and Cytokines

**DOI:** 10.3390/biomedicines11092431

**Published:** 2023-08-31

**Authors:** Lina Lietuvninkas, Basma Baccouche, Andrius Kazlauskas

**Affiliations:** 1 Department of Ophthalmology and Visual Sciences, University of Illinois at Chicago, Chicago, IL 60612, USA; lel2@uic.edu (L.L.);; 2 Department of Physiology and Biophysics, University of Illinois at Chicago, Chicago, IL 60612, USA

**Keywords:** RepSox, endothelial cells, permeability, diabetes, retinopathy, anti-VEGF, cytokines

## Abstract

The therapeutic benefit provided by anti-vascular endothelial growth factor (VEGF) for patients with vision-threatening conditions such as diabetic retinopathy (DR) demonstrates the important role of VEGF in this affliction. Cytokines, which can be elevated in the vitreous of patients with DR, promote leakage of retinal blood vessels, and may also contribute to pathology, especially in those patients for whom anti-VEGF does not provide adequate benefit. In this in vitro study using primary human retinal endothelial cells, we compared anti-VEGF with the (transforming growth factor beta) TGFβ receptor inhibitor RepSox (RS) for their ability to enforce barrier function in the face of VEGF, cytokines, and the combination of both. RS was superior to anti-VEGF because it prevented permeability in response to VEGF, cytokines, and their combination, whereas anti-VEGF was effective against VEGF alone. The inhibitory effect of RS was associated with suppression of both agonist-induced pore formation and disorganization of adherens junctions. RS-mediated inhibition of the TGFβ pathway and increased expression of claudin-5 did not adequately explain how RS stabilized the endothelial cell barrier. Finally, RS not only prevented barrier relaxation, but also completely or partially reclosed a barrier relaxed with tumor necrosis factor α (TNF α) or VEGF, respectively. These studies demonstrate that RS stabilized the endothelial barrier in the face of both cytokines and VEGF, and thereby identify RS as a therapeutic that has the potential to overcome permeability driven by multiple agonists that play a role in the pathology of DR.

## 1. Introduction

Diabetic retinopathy (DR) is a complication that can develop in the eyes of patients with diabetes mellitus (DM) [1]. The diabetic milieu within the circulation damages the retinal vasculature and thereby increases the vitreal concentration of growth factors (such as vascular endothelial growth factor (VEGF)) and cytokines (such as tumor necrosis factor α (TNFα) and interleukin-1 beta (IL-1β)). Elevated levels of such agents drive blood–retinal barrier (BRB) breakdown and neovascularization—diagnostic features of advanced DR [2].

While intensive blood glucose management may delay the onset of DR, most patients with DM eventually develop DR [3]. Reducing the vitreal level of VEGF by intravitreal administration of anti-VEGFs has become the standard of care for patients with DR [4]. Not all patients experience an adequate therapeutic benefit from anti-VEGF treatment, perhaps because of elevated levels of cytokines, which are unaffected by anti-VEGFs [2,3]. Antagonizing both VEGF and cytokines is expected to be effective for a greater percentage of patients with DR.

RepSox (RS) is a small-molecule multi-kinase inhibitor that prevents VEGF-induced permeability [5]. Because one of the RS targets is TGF-βR1 [6], and the TGFβ family governs vascular homeostasis [7], it is possible that TGF-βR1 is an essential mediator of VEGF-induced permeability. However, not all TGF-βR1 inhibitors prevent VEGF-driven barrier relaxation [5], which suggests that the relevant RS target is a kinase other than TGF-βR1.

In this report we investigated the therapeutic potential of RS. We considered whether RS prevents both cytokine- and VEGF-induced permeability of primary human retinal endothelial cells (HRECs), its mechanism of action, and its ability to reclose a relaxed barrier. Finally, we performed a head-to head comparison of RS and an anti-VEGF (aflibercept).

## 2. Materials and Methods

### 2.1. Materials

Primary human retinal endothelial cells (HRECs) were purchased from Cell Systems (Kirkland, WA, USA). They were cultured in EBM^TM^-2 Basal Medium (CC-3156) supplemented with EGM^TM^-2 MV Microvascular Endothelial Cell Growth Medium SingleQuots^TM^ (CC-4147) purchased from Lonza Biosciences (Verviers, Belgium). D-(+)-glucose (G7021) and 2% gelatin solution (G1393) were purchased from Sigma Aldrich (St. Louis, MO, USA). Corning™ Penicillin–Streptomycin Solution (MT30002CI) was purchased from ThermoFisher Scientific (Waltham, MA, USA).

RS was purchased from both Selleckchem (S7223) and Cayman (14794). Recombinant human VEGF 165 (100-20), recombinant human TNFα (300-01A), recombinant human IL-1β (200-01B), and recombinant human TGFβ1 (100-21) were from Peprotech, Inc. (Cranbury, NJ, USA). Aflibercept (Eylea) was from Regeneron Pharmaceuticals Inc. (Tarrytown, NY, USA). ON-TARGETplus human SMAD4 siRNA (L-003902-00-0005), ON-TARGETplus Non-targeting Control Pool (D-001810-10-20), and DharmaFECT 1 Transfection Reagent (T-2001-03) were purchased from Horizon Discovery, Ltd. (Waterbeach, UK). Opti-MEM™ reduced serum medium (31985070) was purchased from ThermoFisher. The 8-well chamber slides for ECIS (8W10E+) were purchased from Applied Biophysics, Inc. (Troy, NY, USA).

Dulbecco’s Phosphate-Buffered Salt Solution 1× (MT21030CM), EZ-Link™ NHS-LC-LC-Biotin (21343), Pierce™ 16% Formaldehyde (28908), and Streptavidin—Alexa Fluor™ 594 Conjugate (S32356) were purchased from ThermoFisher Scientific. Bovine serum albumin (BSA (A2153-50G)), and DAPI (D9542-10MG) were purchased from Sigma Aldrich. Human VE-cadherin antibody (MAB9381-SP) was purchased from R&D Systems (Minneapolis, MN, USA). Goat Anti-Mouse IgG H&L (Alexa Fluor^®^ 488) (ab150113) and normal goat serum (ab7481) were purchased from Abcam (Boston, MA, USA). DAPI Fluoromount-G^®^ (0100-20) was purchased from SouthernBiotech (Birmingham, AL, USA).

SMAD4 polyclonal antibody (PA5-34806) was purchased from ThermoFisher Scientific. Phospho-SMAD3 (Ser423/425) antibody (9520S) and anti-rabbit IgG HRP-linked antibody (7074) were purchased from Cell Signaling, Inc. (Danvers, MA, USA). RasGAP antibody was an antiserum that was raised as described previously [8]. Claudin-5 monoclonal antibody (35–2500) was purchased from ThermoFisher. IRDye^®^ 800CW Donkey anti-Mouse IgG secondary antibody (926–32,212) and Goat anti-Rabbit IgG secondary antibody (926–32,211) were purchased from LI-COR, Inc. (Lincoln, NE, USA).

Precast 7.5%, 10%, and 12% Mini-PROTEAN^®^ TGX™ gels (4561034) were from Bio-Rad Laboratories, Inc. (Hercules, CA, USA). Pierce™ ECL Western Blotting Substrate (PI32106) and iBlot™ 2 Transfer Stacks were purchased from ThermoFisher Scientific.

### 2.2. Cell Culture

HRECs were seeded onto standard polystyrene plates coated with a 0.04% gelatin solution and cultured in Lonza EBM basal media supplemented with EGM SingleQuots and 5% penicillin–streptomycin solution. Cells were cultured in either 5 mM D-glucose-containing media, which we denoted as normal glucose (NG) media, or media supplemented with additional glucose to a final concentration of 30 mM, which we denoted as high-glucose (HG) media. Our in vitro system emulating diabetic retinopathy requires HRECs to be treated with HG for at least 10 days with daily media changes. Experiments commenced after this 10-day treatment with HG.

### 2.3. Electrical Cell-Substrate Impedance Sensing (ECIS)

The transendothelial electrical resistance (TEER) of HRECs was measured using an ECIS ZTheta instrument (Applied Biophysics, Troy, NY, USA) housed within a standard tissue culture incubator, as described previously [9]. First, 17.6 × 10^6^ cells were plated onto gelatin-coated 8-well chamber slides containing gold-plated microelectrodes (Applied Biophysics catalogue #8W10E+, New York, NY, USA). The following day, each well was inspected under a phase-contrast microscope to verify that the monolayer was complete and overtly normal. At the start of the experiment (typically 24–48 h after plating), the impedance was typically 1500 ohms at 16 kHz. Four wells of the TEER chamber were used for a single experimental condition.

After placing the chamber in the TEER instrument, the impedance was monitored until it stabilized (typically at least 30 min). The electric current passing through the endothelial monolayers was measured independently in each well. TEER was measured continuously and in real time before, during and after the treatment of cells. When the medium was changed during a measurement period, the recording was paused until the medium change-induced noise subsided.

TEER data are presented in ohms without normalization. The starting impedance was typically very similar for all wells; variants, wells in which the impedance was ±15% of the average of all of the wells, were not included. The TEER tracing shows the mean ± SEM of the four wells of a given experimental condition. The area under the curve (AUC) of data sets was quantified using ImageJ (version: 2.0.0-rc-69/1.52v; build: 269a0ad53f; date: 2018-12-04; open source image processing software; https://imagej.net/software/fiji; Bethesda, MD, USA). Unless indicated otherwise, the TEER tracing is of a single, representative experiment, whereas the AUC data are the compilation of all independent experiments within a series. The AUC data, determined from the TEER tracing for the same time period for all experimental conditions, were expressed as a ratio of the agent-treated/vehicle-treated samples. The bar graph shows the mean ± SEM of the agent-treated/vehicle-treated ratio for all of the independent experiments within a series.

For experiments testing the ability of RS to prevent agonist-induced barrier relaxation, 10 µM RS was added, followed by the immediate addition of 100 ng/mL VEGF and/or 50 ng/mL TNFα or 50 ng/mL IL-1β. For RS and aflibercept reclosure experiments, cells were exposed to 100 ng/mL VEGF and/or 50 ng/mL TNFα for 8 h before the addition of either 10 µM RS or 143.75 µg/mL aflibercept for the remainder of the time course. To reflect that clinical scenario, aflibercept was added at a 500-fold molar excess of VEGF (1250 nM aflibercept and 2.5 nM VEGF). The composition of the aflibercept vehicle was 10 mM sodium phosphate pH 6.2, 40 mM sodium chloride, 0.03% polysorbate 20, and 5% sucrose. In this experimental system, bevacizumab, which neutralizes VEGF-A, had the same effect as aflibercept, which neutralizes VEGF-B and PIGF in addition to VEGF-A [9].

### 2.4. Gelatin Trapping Assay (GTA)

The formulation of biotinylated gelatin and the gelatin trapping assay (GTA) protocol was as previously described [10]. Briefly, cells were seeded at 100% confluency on a 35 mm glass bottom dish (P35G-1.5-14-C) from MatTek Corporation (Ashland, MA, USA) that had been coated with 100 µL of 0.25 µg/mL biotinylated gelatin overnight at 4 °C. The medium was refreshed every 24 h; 48 h post-plating, 10 µM RS or vehicle, 100 ng/mL VEGF, and 50 ng/mL TNFα or vehicle were added and incubated for 5 h. Once stimulation was complete, cells were treated briefly with a 1:2000 dilution of streptavidin in DPBS. The remaining protocol was completed in the dark using 1000 µL of treatment solution in every step. Cells were washed three times with room-temperature DPBS and treated with 4% PFA in DPBS for 10 min at room temperature. After three 5 min washes with ice-cold DPBS, cells were treated with permeabilization solution (0.25% TritonX100 in DPBS) for 30 min at room temperature. Once cells were permeabilized, they were washed again with DPBS three times for 5 min per wash. Plates were blocked with AD buffer (10% goat serum, 1% BSA, 0.05% Tween20 in DPBS) for 1 h at room temperature. After aspirating the AD buffer, cells were incubated overnight at 4 °C with a 1:200 dilution of VE-cadherin primary antibody in AD buffer. The next day, the cells were washed with DPBS containing 0.05% Tween 20 (PBST) three times for 5 min per wash, and then incubated for 1 h at room temperature with a 1:1000 dilution of fluorescently tagged secondary antibody in AD buffer. Once incubation with the secondary antibody was complete, the cells were washed two times in PBST and once in PBS for 5 min per wash, and then counterstained with DAPI diluted in DPBS 1:1000 for one minute. After one final wash with DPBS, 12 mm glass coverslips were mounted onto the glass bottom using a drop of antifade mounting medium.

Plates containing fixed cells were subjected to imaging with a Zeiss confocal microscope (Jena, Germany). At least two images per plate were collected at 20× objective using 405 nm (DAPI), 488 nm (VE-cadherin), and 594 nm (streptavidin) lasers. Quantification of pore intensity was accomplished using ImageJ.

### 2.5. Small Interfering RNA (siRNA)

HG HRECs were plated onto 60 mm gelatin-coated plates and grown to confluency in complete Lonza medium without antibiotic supplements. Cells were transfected with 2 mL of 10 nM non-targeting control or siSMAD4 complexed with Dharmacon transfecting reagent (1:2 ratio) in Opti-mem medium. The medium was replaced with complete Lonza medium 24 h post-transfection and incubated for another 24 h (total of 48 h of knockdown) before sample collection for Western blot analysis.

For ECIS analysis, cells were plated to confluency on two 100 mm gelatin-coated dishes cultured in Lonza medium without antibiotic supplements. Cells were transfected with 4 mL of 10 nM siSMAD4 or 10 nM non-targeting control complexed with Dharmacon transfecting reagent (1:2 ratio) in Opti-mem medium for 24 h. After this incubation, cells were plated on gelatin-coated 8-well chamber slides for ECIS (8W10E+); 24 h later (after 48 h of knockdown), the ECIS assay began in which 100 ng/mL VEGF or vehicle was added to the wells. TEER tracings were recorded for at least 20 h.

### 2.6. Western Blotting

HG HRECs were plated onto 60 mm or 35 mm gelatin-coated plates and grown to confluency before treatment with 10 µM RS or vehicle for 30 min, followed by 50 ng/mL TGFβ or vehicle for an additional 30 min. Cells were then washed with ice-cold PBS and lysed in SDS-βMe (0.3% sodium dodecyl sulfate, 1% β-mercaptoethanol, and 50 mM Tris-HCl pH 7.5). Lysates were then treated with DNase/RNase (1 mg/mL DNase I, 500 µg/mL RNaseA, 100 mM Tris, pH 7.5, 25 mM MgCl2, and 5 mM CaCl2) and 5× sample buffer (10 mM EDTA, 2% sodium dodecyl sulfate, 0.2 M 2-mercaptoethanol, 20% glycerol, 20 mM Tris-HCl, pH 6.8, and 0.2% bromophenol blue) before being heated at 95 °C for 5 min to denature the proteins. Samples were then cooled on ice (or stored at −20 °C) before being resolved on a 7.5% SDS–polyacrylamide gel and subjected to Western blot analysis. siRNA-transfected cells and RS (10 µM)-stimulated cells were collected in the same way and resolved on 10% and 12% SDS–polyacrylamide gels, respectively.

After transferring the gel using iBlot stacks, membranes were incubated on an orbital shaker for 1 h in TBST (10 mM Tris, pH 7.5, 150 mM NaCl, and 0.05% Tween20) containing 5% bovine serum albumin (BSA) at room temperature. Blots were subsequently incubated with primary antibodies (SMAD4 (1:1000), pSMAD3 (1.3:1000) claudin-5 (1:1000), and Rasgap (1:2000)) at room temperature for 2–3 h or overnight at 4 °C before washing and probing with secondary antibody for 1 h at room temperature. An HRP-linked secondary antibody was used for the SMAD4 and pSMAD3 blots, and IRDye 800CW secondary antibodies were used for the claudin-5 blots. ECL substrate kits were used to visualize the SMAD4 and pSMAD3 blots. The claudin-5 blots were visualized with the Chemidoc MP imager. The intensity of the bands was quantified using ImageJ.

### 2.7. Statistical Analysis

Unless indicated otherwise, TEER data are mean ± SEM; all other data are mean ± SD. Statistical significance of differences between means of two experimental groups was assessed using the *t*-test; ANOVA was used when comparisons involved groups larger than two. Significance was defined as *p* < 0.05.

## 3. Results

### 3.1. RS Prevented Barrier Relaxation by Distinct Agonists

**Figure 1:**
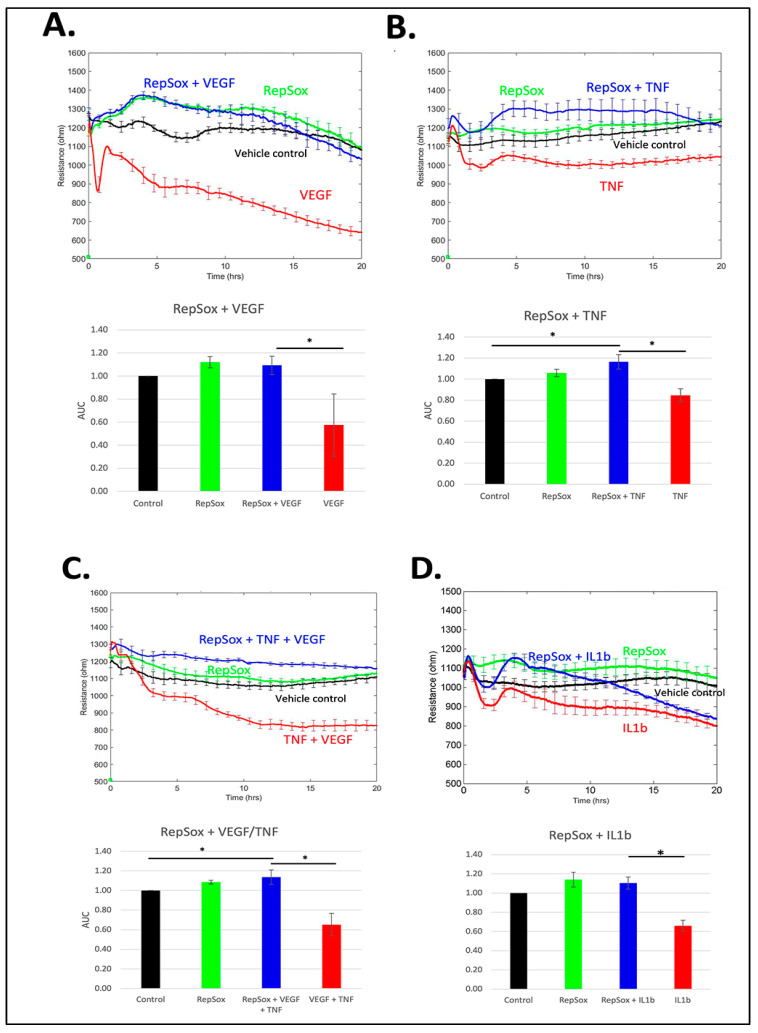
RS prevented barrier permeability by distinct agonists. TEER tracings of cells treated with either DMSO or 10 µM RS that was added concurrently with agonist vehicle (0.1% BSA in water) or (**A**) 100 ng/mL VEGF, (**B**) 50 ng/mL TNFα, (**C**) 100 ng/mL VEGF and 50 ng/mL TNFα, or (**D**) 50 ng/mL IL-1β. The TEER tracings from a single, representative experiment are shown; the data are the mean ± SEM of 4 wells that were used for a single experimental condition. The bar graphs show the average area under the curve (AUC) from three independent experiments. The AUC was calculated for the time interval from 0 to 15 h. Differences between the indicated pairs were determined using the Student’s *t*-test; * *p* < 0.05.

We investigated the effect of RS on both basal and agonist-induced permeability in primary human retinal endothelial cells (HRECs) using electric cell-substrate impedance sensing (ECIS), which measures the electrical resistance across a monolayer of cells. Addition of RS rapidly improved basal barrier function and completely prevented VEGF from relaxing the barrier (Figure 1A). Dose–response experiments indicated that 10 µM was the minimum concentration of RS that had a maximal effect; 1 and 0.1 µM had a partial effect and no effect, respectively. These observations resonate with a previous publication investigating the influence of a panel of TGFβ receptor antagonists on basal and VEGF-induced permeability [5]. We extended this line of investigation by considering the effect of RS on cytokine-induced permeability. The effect was even more pronounced. RS not only prevented TNFα or IL-1β permeability, but it also converted TNFα from a barrier relaxer to a modest barrier enforcer (Figure 1B,D). Finally, RS prevented relaxation in response to the combination of TNFα and VEGF (Figure 1C). These studies demonstrate that RS enforces basal barrier function and prevents relaxation in response to multiple types of agonists.

The experiments in Figure 1 were conducted with HRECs cultured in high glucose (30 mM). We recently reported that these experimental conditions induced hyperglycemia-induced adaptation (HIMA) [11]. To test if the effects of RS described above were unique to this in vitro model of diabetic retinopathy, we repeated key experiments with HRECs cultured in normal glucose (5 mM). The effect of RS was the same in cells that had not undergone HIMA (Figure S1). Thus, HG-induced changes to gene expression, osmolality, and metabolism did not influence the response of cells to RS. We conclude that acquisition of HIMA was not required for the RS effect, which is consistent with the report of Roudnicky et al. using cells cultured in normal glucose [5].

### 3.2. Antagonizing TGFβ Signaling Was Insufficient for the RS Effect

**Figure 2:**
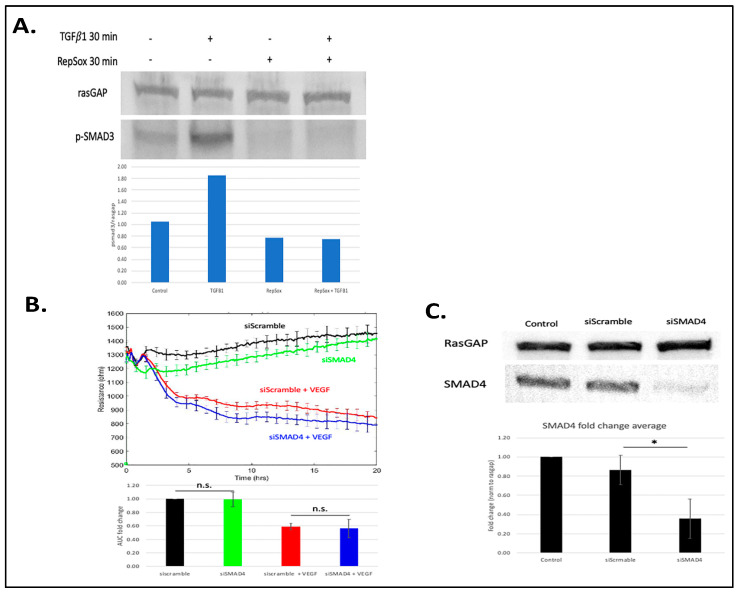
RS’s inhibition of TGFβ-induced signaling was not responsible for its effect on agonist-induced permeability. (**A**) Cells were pretreated with RS vehicle (DMSO) or 10 µM RS for 30 min before adding TGFβ vehicle (0.1% BSA in water) or 50 ng/mL TGFβ for an additional 30 min, after which time the cells were lysed and subjected to Western blot analysis with the indicated antibodies. Band intensities were quantified using ImageJ. The pSMAD3/Rasgap ratio for the blot shown is presented in the bar graph. Similar results (efficient RS-mediated suppression of the TGFβ pathway) were observed on at least three independent occasions. (**B**) Same as Figure 1A, except the cells were transfected with either siScramble or siSMAD4. The TEER tracing is of a single, representative experiment; the bar graph is the compilation of at least three independent experiments. (**C**) The cells used in panel (**B**) were subjected to Western blot analysis using the indicated antibodies. The blot is of a single, representative experiment, while the bar graph shows the fold change in the intensity of SMAD4 in three independent experiments. * *p* < 0.05; n.s. *p* > 0.05.

**Figure 3:**
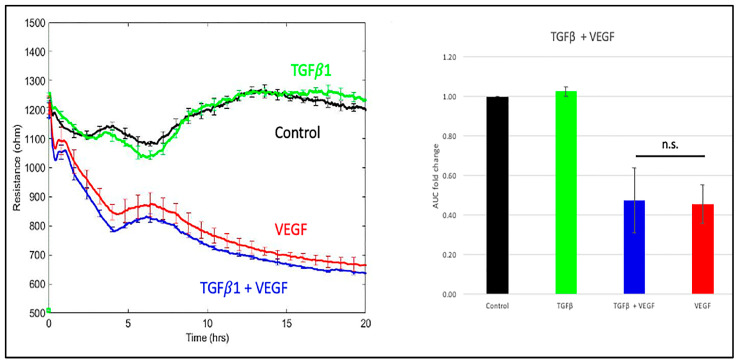
Overnight treatment of TGFβ had no effect on VEGF-induced permeability. Cells were pretreated overnight with 50 ng/mL TGFβ or TGFβ vehicle (0.1% BSA in water), and then 100 ng/mL VEGF or PBS (control) was added, and the TEER tracing was recorded for the indicated duration. The TEER tracing from a single, representative experiment is shown; the data are the mean ± SEM of the 4 wells that were used for a single experimental condition. The bar graphs show the mean ± SD of the area under the curve (AUC) from three independent experiments. The AUC was calculated for the time interval from 0 to 20 h. Differences between the indicated pairs were determined using the Student’s *t*-test; n.s. *p* > 0.05.

In light of the well-established ability of RS to inhibit the kinase activity of TGFβ receptors [5,12], we sought to test the hypothesis that RS enforced basal barrier function and prevented agonist-induced relaxation by antagonizing constitutive TGFβ signaling. We found that while RS inhibited TGFβ-induced signaling (Figure 2A), constitutive TGFβ signaling (phosphorylation of SMAD3) was low (Figure 2A; see also the uncropped blot in Figure S2. Furthermore, antagonizing the endogenous TGFβ pathway by suppressing the expression of SMAD4 did not phenocopy the RS effect (Figure 2B,C; see also the uncropped blot in Figure S2). In addition, exposing cells to exogenous TGFβ for either 18 or 48 h had no effect on basal or VEGF-induced permeability (Figure 3 and [13]). These data show that antagonizing or activating the TGFβ pathway did not influence basal or VEGF-mediated barrier relaxation. Moreover, although RS prevented TGFβ signaling, this did not appear to be how it enforced barrier function. This concept is supported by Roudnicky et al., showing that TGFβ receptor inhibitors other than RS (e.g., SB-431542) had no effect on barrier function. This unique feature of RS appears to require more than suppression of TGFβ-dependent signaling.

### 3.3. RS Prevented Agonist-Induced Pore Formation and VE-Cadherin Disorganization

To better understand how RS enforced barrier function, we investigated its influence on pore formation and the organization of adherens junctions, which are components of the barrier that are affected by many types of agonists [14]. To observe pore formation, we used a previously described gelatin trapping assay (GTA) [10] in which cells are plated on biotinylated gelatin and stained with fluorescently labeled streptavidin. Pores permit interaction between biotin and streptavidin, which can be detected via fluorescent microscopy and quantified. The organization of adherens junctions was assessed by co-staining the monolayers with a fluorescently labeled anti-VE-cadherin antibody. Control experiments demonstrated the specificity of the signals that were observed in this series of experiments (Figure S3).

**Figure 4:**
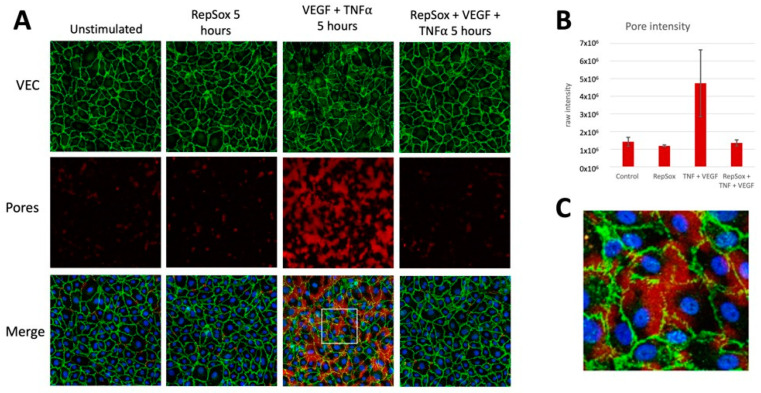
RS prevented agonist-induced pore formation and VEC disorganization. (**A**) Cells that had been treated with the indicated agents were subject to the GTA assay as described in the Materials and Methods section. The top row of this panel shows staining with an anti-VE-cadherin antibody, which reveals the nature of the adherens junction. (**B**) Quantification of pore intensity (red signal). The bars are the median of the intensity from two arbitrarily selected images; error bars show the range. In 3 independent experiments, RS inhibited VEGF/TNFα-induced pore formation. (**C**) High magnification of the boxed region within the merged VEGF and TNFα image (the third panel from the right in the bottom row). This panel demonstrates that pores formed preferentially in regions of VEC disorganization.

Exposing cells to the combination of VEGF and TNFα for 5 h increased permeability (Figure 1C) induced pore formation and disorganized the adherens junctions (Figure 4). A high-magnification image shows that pores preferentially formed in areas where the adherens junctions had disorganized (Figure 4). RS prevented all of these changes (Figure 1C and Figure 4). While RS improved the electrical resistance of the barrier in unstimulated cells (Figure 1), RS did not detectably affect pores or the organization of the adherens junctions under such experimental conditions (Figure 4). These data indicate that a plausible mechanism by which RS suppresses agonist-induced permeability is by preventing changes in the barrier that are required for its relaxation.

We also considered whether the RS effect was associated with increased expression of claudin-5, a component of tight junctions that contributes to endothelial barrier stability [15]. As expected, and as in [5], expression of claudin-5 was markedly increased following exposure to RS for 24 h (Figure S4; see also the uncropped blot in Figure S5). However, the level of claudin-5 was unchanged in cells incubated with RS for 1 or 6 h (Figure S4; see also the uncropped blot in Figure S5). Because the RS effect was observed very quickly, well before the increase in expression of claudin-5, we conclude that upregulation of claudin-5 is unlikely to be responsible for how RS stabilized the barrier in our experimental setting.

### 3.4. RS Reclosed the Barrier Relaxed by Distinct Agonists

In addition to preventing agonist-induced permeability (Figure 1), we considered whether RS was able to reclose a relaxed barrier. The design of these experiments was to first relax the barrier for 8 h with VEGF, TNFα, or their combination before adding RS and continuing to record the permeability of the monolayers. Thus, we tested if RS could reclose the barrier in the continued presence of the agonist that relaxed it.

**Figure 5:**
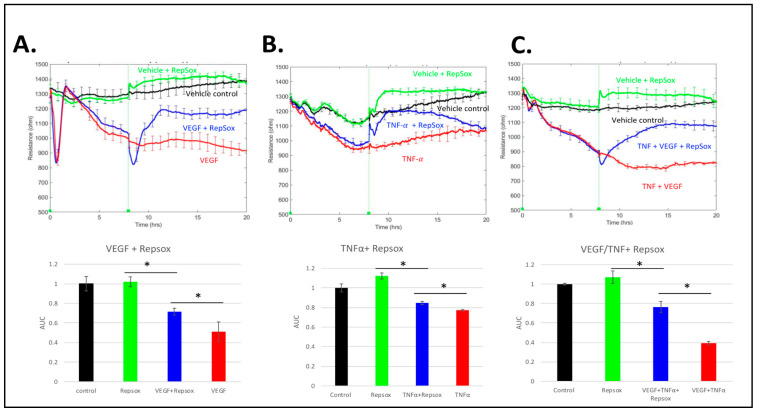
RS reclosed barriers that were relaxed by distinct agonists. Barriers were relaxed with either (**A**) 100 ng/mL VEGF, (**B**) 50 ng/mL TNFα, or (**C**) 100 ng/mL VEGF and 50 ng/mL TNFα for 8 h before the addition of either RS vehicle (DMSO) or 10 µM RS. Each TEER tracing is from a single, representative experiment along with the bar graph showing the area under the curve (AUC) for that experiment, which was calculated for the time interval from 15 to 20 h. The error bars show the mean ± SEM for quadruplicate wells that were used for each experimental condition. The Student’s *t*-test was used to determine statistical significance between the indicated pairs. * *p* < 0.05 Three independent experiments showed similar results. Repeat experiments can be found in Figures S6–S8.

**Figure 6:**
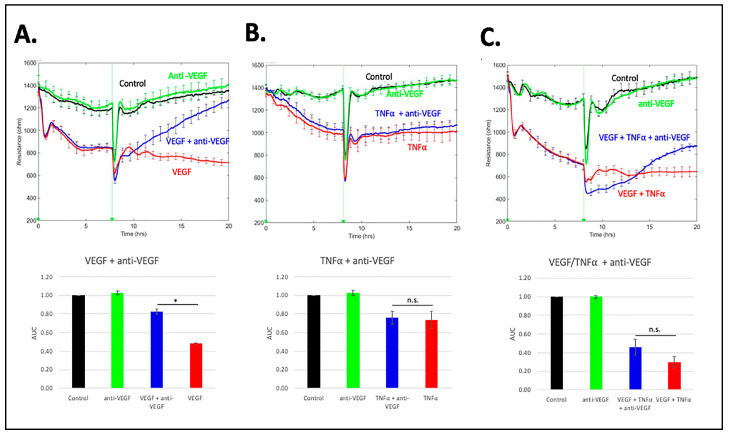
Anti-VEGF reverses only VEGF-mediated barrier relaxation. Same as Figure 5, except aflibercept vehicle or aflibercept was used instead of RS. Barriers were relaxed with either (**A**) 100 ng/mL VEGF, (**B**) 50 ng/mL TNFα, or (**C**) 100 ng/mL VEGF and 50 ng/mL TNFα for 8 h before the addition of either aflibercept vehicle or aflibercept (anti-VEGF). TEER tracings are from a single, representative experiment; the data are the mean ± SEM of the 4 wells that were used for each experimental condition. The bar graphs under the TEER tracings show the mean ± SD of the area under the curve (AUC) for the TEER tracing shown above. The AUC was calculated for the time interval from 15 to 20 h. Statistically significant differences between the indicated pairs were determined using the Student’s *t*-test; * *p* < 0.05; n.s. *p* > 0.05. Three independent experiments showed similar results.

RS partially reclosed a barrier relaxed by VEGF, and the maximum effect took approximately 4 h (Figure 5A). In contrast, RS completely and quickly overcame TNFα-induced permeability (Figure 5B). Cells treated with both VEGF and TNFα responded to the addition of RS in a manner similar to cells treated with VEGF alone (Figure 5C). It seems likely that the combination of RS and anti-VEGF treatment would completely reclose the barrier because anti-VEGF is more effective than RS at reclosing a VEGF-relaxed barrier (ref. [9] and Figure 6 below). These results indicate that RS induced reclosure of the barrier in the continued presence of the agonist which relaxed it. Furthermore, the extent of this effect was determined by the agonist.

### 3.5. Comparison of RS with Anti-VEGF

In order to compare RS with anti-VEGF, we repeated the experiments described in Figure 5 using an anti-VEGF (aflibercept) instead of RS. Anti-VEGF reclosed the barrier relaxed with VEGF (Figure 6A) to a greater extent than RS (Figure 5A). Anti-VEGF had no effect on the TNFα -relaxed barrier (Figure 6B), whereas RS completely reclosed it (Figure 5B). Permeability driven by the combination of TNFα and VEGF was largely insensitive to anti-VEGF (Figure 6C), whereas RS partially reclosed a barrier that had been relaxed by this agonist combination (Figure 5C). We conclude that RS either completely or partially overcame permeability driven by different types of agonists. In contrast, anti-VEGF was effective only when VEGF was the sole agonist; under such conditions, it was more effective than RS.

## 4. Discussion

We observed that the therapeutic potential of RS extended beyond the previously reported inhibition of VEGF-induced permeability [5]. RS completely suppressed cytokine (TNFα or IL-1β)-induced and even VEGF/TNFα-induced barrier relaxation. Furthermore, RS either partially (VEGF) or completely (TNFα) reclosed barriers, despite the continued presence of the relaxation-promoting agent. Finally, a head-to head comparison with anti-VEGF indicated that RS stabilized barriers in the face of multiple types of agonists (VEGF and cytokines), whereas anti-VEGF was effective when VEGF was the only agonist present.

The results described herein, together with those published by other groups [12,16,17,18], raise the question of how RS enforces barrier stability. While we observed the expected RS-mediated inhibition of the TGFβ pathway, this did not appear to suffice in our experimental system. Activating or suppressing the TGFβ pathway did not influence either basal or agonist-driven permeability [13] (Figure 2 and Figure 3). Furthermore, other TGFβ pathway inhibitors (SB-431542) do not prevent VEGF-induced permeability [5]. While these observations do not rule out a contribution of the TGFβ pathway, they show that additional targets play an essential role in the effect of RS on barrier function.

Given that RS prevented agonists that engage distinct signaling pathways, it seems most likely that the relevant RS target lies within steps controlling the barrier that are common to multiple agonists (e.g., VEGF, TNFα, and IL1β) [19]. Indeed, RS prevented pore formation and adherens junction disorganization driven by the combination of VEGF and TNFα (Figure 4). While these data are consistent with the RS target being involved with control of components of the barrier, they do not rule out the possibility that the target is proximal to the receptor. For instance, candidate RS targets exist among the kinases that are involved with clathrin-mediated internalization [5], a process that both VEGF and TNFα receptors require for proper signal transduction [20,21]. It is plausible that RS simultaneously antagonizes many kinases, and that inhibition of more than one of them is required for the RS effect.

Is it likely that the relevant RS-regulated kinases govern expression of claudin-5? Claudin-5 is a key component of tight junctions, and its expression declines in response to agents that induce permeability [22]. Furthermore, RS increases the level of claudin-5, and this change correlates with RS-mediated barrier stabilization [23]. However, while RS increased expression of claudin-5 in HRECs, this occurred well after the barrier had been stabilized (Figure S4). Thus, regulation of claudin-5 expression does not adequately explain how RS stabilizes the barrier of HRECs. Additional studies are necessary to identify the RS targets (kinases and pathways that they regulate) responsible for enforcing barrier function.

Unlike anti-VEGF, which physically binds and thereby neutralizes the agonist responsible for destabilizing the barrier, RS prevented cells from responding to the agonist, despite its continued presence. Similarly, RS reclosed a relaxed barrier without neutralizing the agent instructing cells to keep the barrier open. This was especially dramatic with TNFα-treated cells, in which case RS completely reclosed the barrier within minutes (Figure 5B). The rapid and complete reclosure of the barrier raises the intriguing possibility that RS engages an as yet-undescribed pathway that instructs cells to close a relaxed barrier.

The next steps to develop RS as a therapeutic include determining the best way to deliver it (eye drops, slow-release depots implanted into the eye, etc.), its pharmacokinetics, and its safety/toxicity. Investigators focused on outcomes other than permeability have reported the feasibility of treating experimental animals with RS [16,18,24]. Given that RS not only prevents agonist-induced permeability but also tightens the basal barrier, it will be particularly important to assess the effect of RS on kidney function, which depends on the relative high permeability of the glomerular vasculature. Additional considerations include the spectrum of RS targets, especially within the TGFβ family.

## 5. Conclusions

RS stabilized the endothelial barrier in the face of both cytokines and VEGF. Consequently, RS is a candidate therapeutic because of its capability of overcoming permeability driven by multiple agonists that play a role in the pathology of DR.

## Data Availability

The data provided herein are available upon request from the corresponding author.

## Supplementary Materials

The following supporting information can be downloaded at: https://www.mdpi.com/article/10.3390/biomedicines11092431/s1. Figure S1: The effect of RS was independent of HIMA acquisition. Figure S2: Uncropped western blot images from Figure 2A with molecular weight standards. Figure S3. Controls for the GTA shown in Figure 4. Figure S4. Claudin-5 expression increases after 24-h of RS treatment. Figure S5. Uncropped western blot images from Figure S3 with molecular weight standards. Figure S6. Experimental repeats for Figure 5A. Figure S7. Experimental repeats for Figure 5B. Figure S8. Experimental repeats for Figure 5C.
