# Supplementary material for: The Multi-Kinase Inhibitor RepSox Enforces Barrier Function in the Face of Both VEGF and Cytokines
Source: Biomedicines. 2023 Aug 31;11(9):2431. doi: 10.3390/biomedicines11092431 (PMC10525881; doi:10.3390/biomedicines11092431)
Supplement: Supplementary file 1 [file biomedicines-11-02431-s001.zip › biomedicines-2509100-supplementary.pdf]

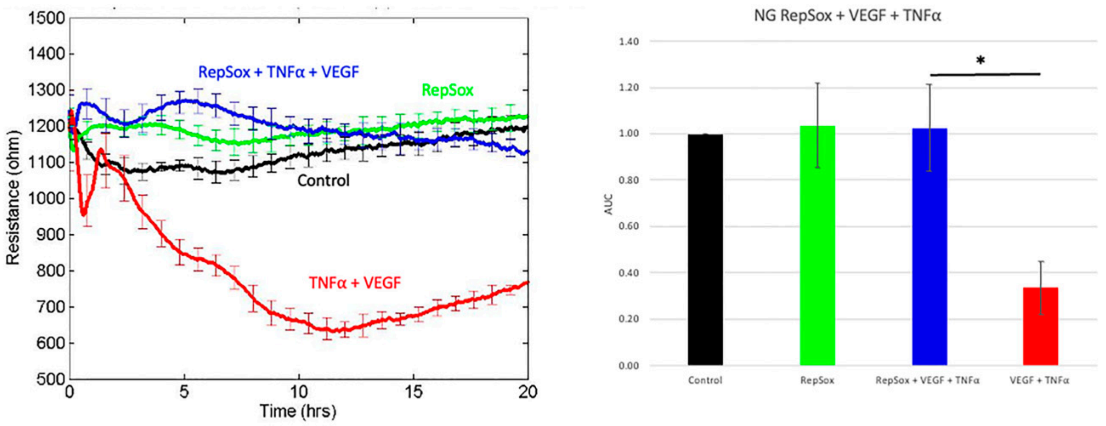

Figure S1. The effect of RS was independent of HIMA acquisition. Same as Figure 1C, except NG cells (cultured in 5 mM) were used instead of HG cells (cultured in 30 mM glucose).

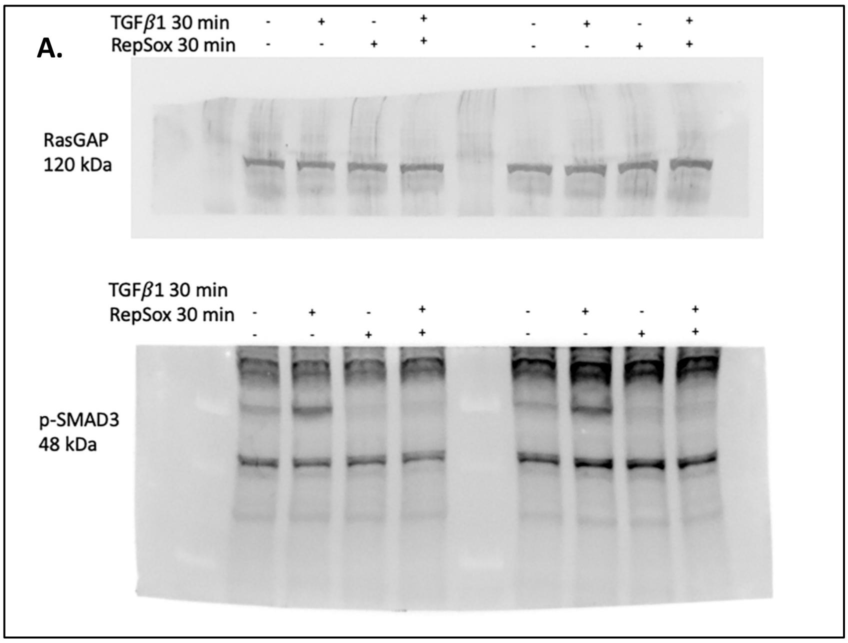

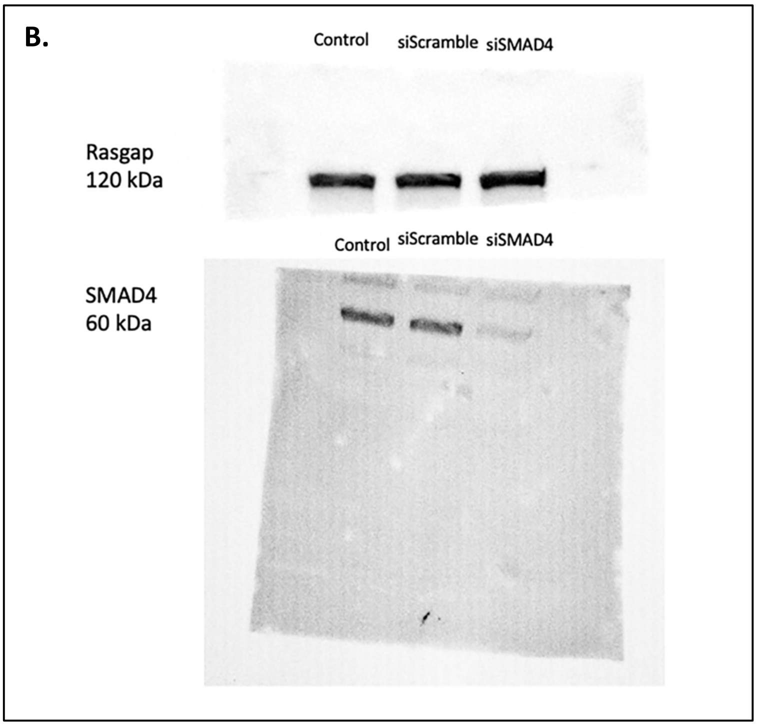

Figure S2. Uncropped western blot images from Figure 2A with molecular weight standards. (A) The images shown are a representative blot indicating the change in SMAD3 activation in RS- and TGFβ1- treated cells. Two independent experiments were processed on the same gel. (B) The images presented are of a representative blot indicating the change in SMAD4 expression after siRNA silencing.

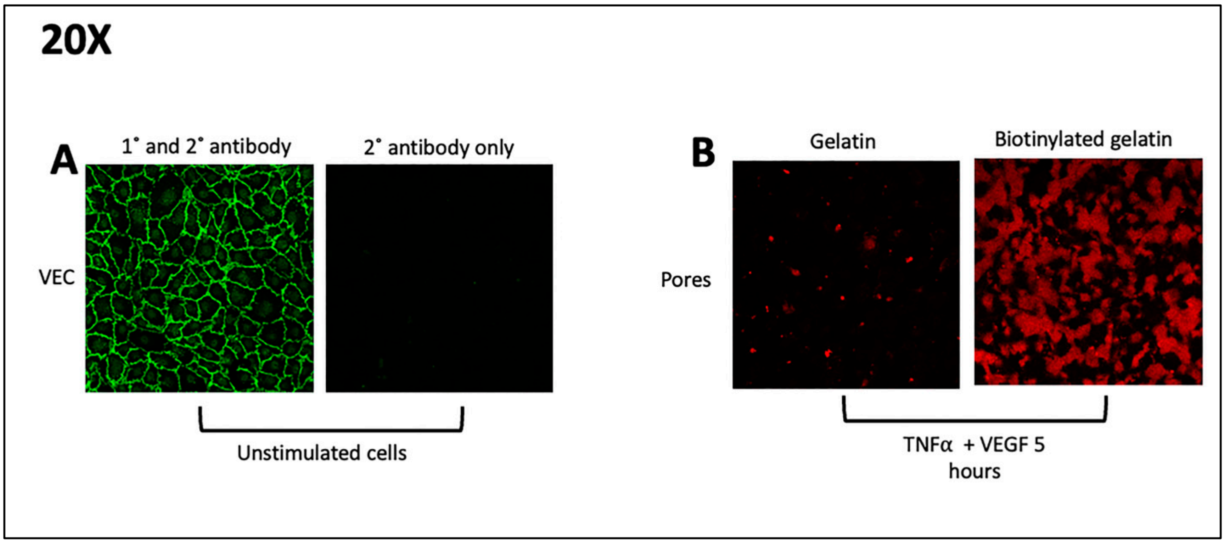

Figure S3. Controls for the GTA shown in Figure 4. Control plates are provided to demonstrate the specificity and effectiveness of the GTA and immunofluorescence staining. (A) Unstimulated cells were stained with or without the anti- VE-cadherin primary antibody and both were stained with the secondary, fluorescently tagged antibody. The image on the left is the same as the top left image in Figure 4. (B) Cells plated on either gelatin, or biotinylated gelatin were stimulated with both TNFα and VEGF for 5 hours and treated with streptavidin containing PBS. The image on the right is the same panel shown in the middle column, third row of Figure 4.

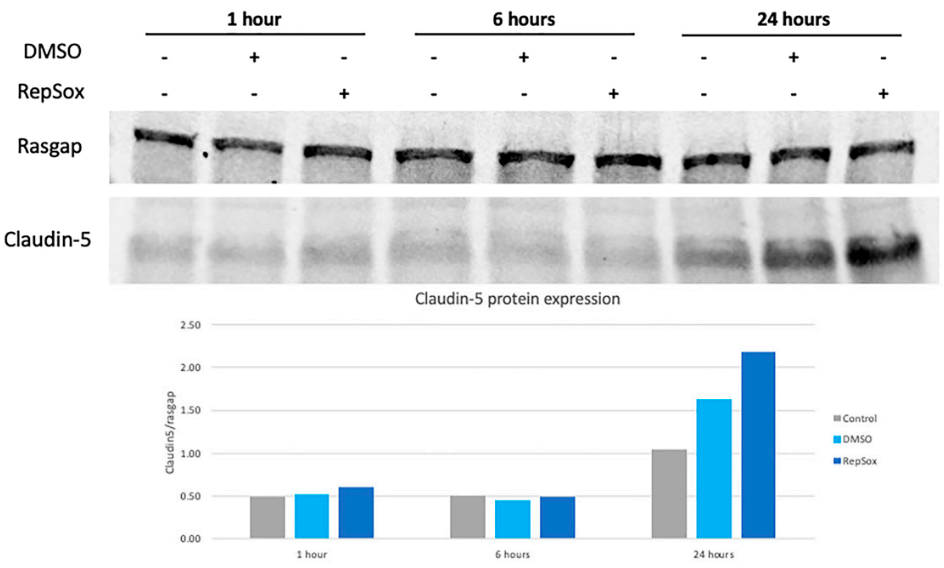

Figure S4. Claudin-5 expression increases after 24-hours of RS treatment. Cells were left resting, exposed to RS vehicle (DMSO) or RS (10 µM) for either 1, 6 or 24 hours, then lysed and subjected to Western blot analysis with the indicated antibodies. The bar graph shows the ratio of the claudin 5/Rasgap signal. Three independent experiments showed similar results.

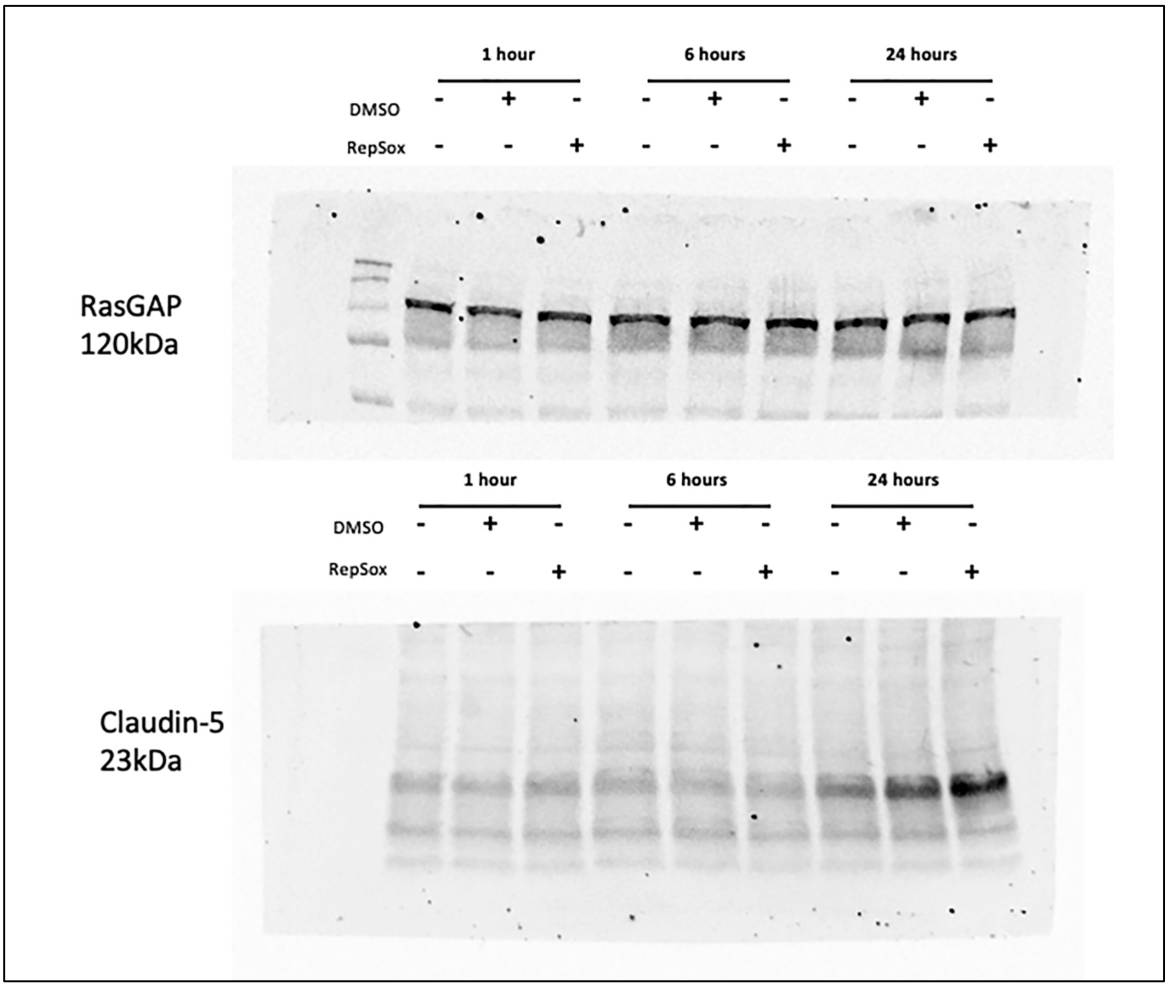

Figure S5. Uncropped western blot images from Figure S3 with molecular weight standards. The images shown are of a representative blot indicating the change in claudin-5 expression in DMSO and RepSox treated cells.

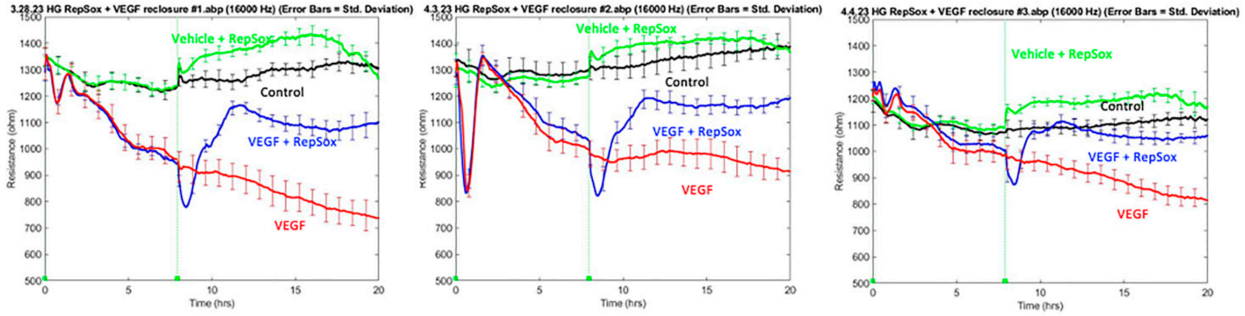

Figure S6. Experimental repeats for Figure 5A.

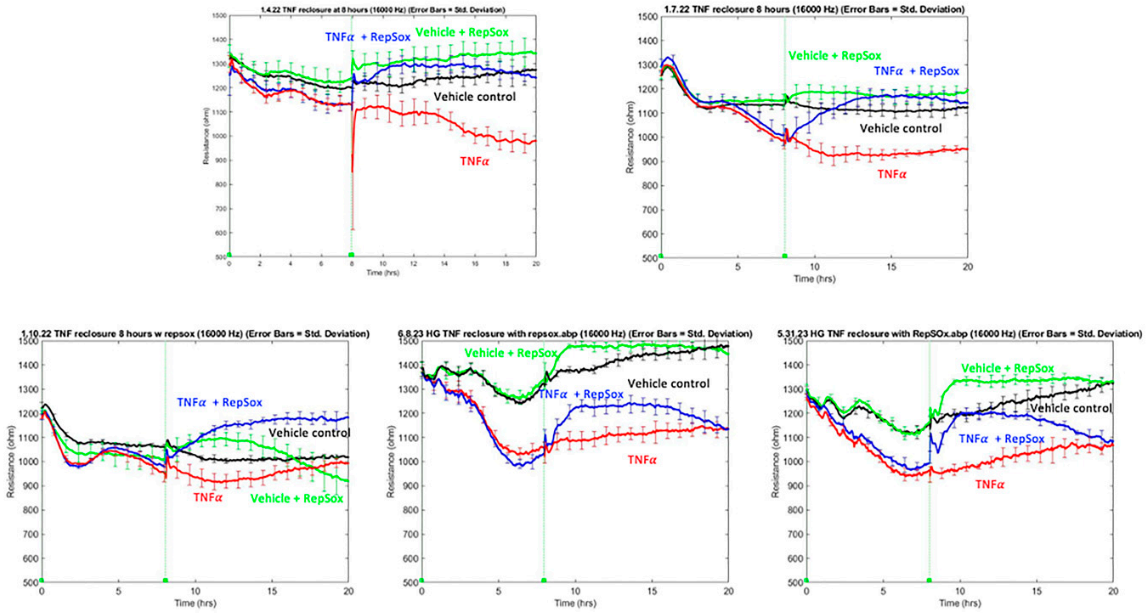

Figure S7. Experimental repeats for Figure 5B.

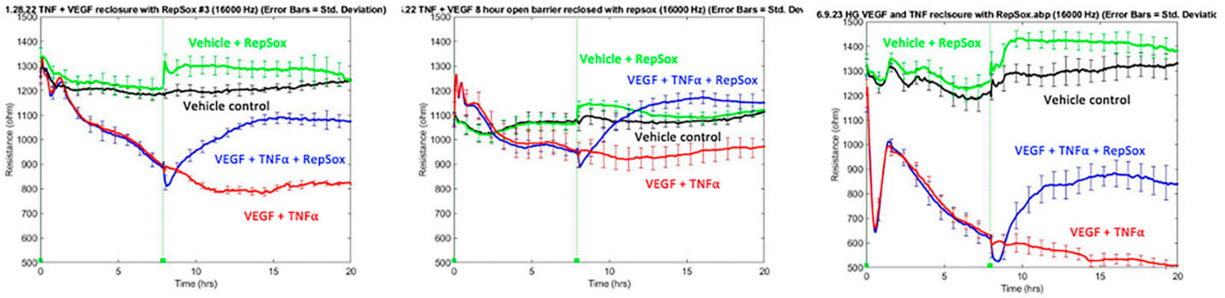

Figure S8. Experimental repeats for Figure 5C.
